# The impact of perceived social support on Chinese university students’ employability: a perspective based on signaling theory

**DOI:** 10.3389/fpsyg.2026.1726553

**Published:** 2026-02-16

**Authors:** Gongjing Wang, Huadi Wang

**Affiliations:** 1 School of Education Science, Nanjing Normal University, Nanjing, China; 2 School of Geography and Planning, Chizhou University, Chizhou, China; 3 Institute of Vocational Education and Lifelong Education of Jiangsu Academy of Educational Sciences, Nanjing, China

**Keywords:** employability, occupational self-efficacy, perceived social support, personal brand equity, signaling theory

## Abstract

**Background:**

Perceived Social Support (PSS) refers to how much an individual subjectively feels cared for, assisted, and supported by others or social groups. It focuses on the individual’s psychological experience and evaluation of support, rather than just the objectively existing supportive behaviors. According to signaling theory, an individual’s perception of social support can act as a positive signal about oneself and the situation, aiding in the acquisition of more effective information and enhancing one’s willingness and ability to adapt during career development. This paper, grounded in signaling theory, investigates how Perceived Social Support influences the mechanisms that shape an individual’s employability. It also analyzes its theoretical role in this process to construct explanatory paths and research propositions.

**Methods:**

This study investigates the impact mechanism of PSS on individual employability through the lens of signaling theory. A cross-sectional survey involving 534 university students in China utilized the Perceived Social Support Scale, Occupational Self-Efficacy Scale, Personal Brand Equity Scale, and Employability Scale, with structural equation modeling employed to analyze the interrelationships among these variables.

**Results:**

The results reveal that: (1) Perceived social support (PSS), occupational self-efficacy (OS-E), personal brand equity (PBE), and employability are significantly and positively correlated; (2) Path analysis demonstrates that perceived social support impacts employability through the mediation of occupational self-efficacy and personal brand equity: the direct mediating effect of OS-E (indirect effect = 0.164), the direct mediating effect of PBE (indirect effect = 0.036), and their sequential mediation (indirect effect = 0.140).

**Conclusion:**

This study, based on a sample of Chinese college students, reveals that perceived social support not only directly predicts employability but also indirectly influences it through a dual-mediation mechanism involving occupational self-efficacy (psychological path) and personal brand equity (behavioral path). Occupational self-efficacy serves as the foundation for accumulating personal brand equity. The findings offer empirical support from the Chinese context for signaling theory in the employment field, demonstrating that the social support system plays a role in shaping and transmitting signals. Furthermore, the study emphasizes the importance of focusing on the collaborative transformation of psychological and behavioral signals in developing employability.

## 1 Introduction

In today’s era of boundaryless careers, the uncertain social environment has heightened employees’ career insecurity (Zyberaj and Bakac, 2022). Individuals must take charge of their careers, adapting to the evolving work landscape by boosting their employability. Currently, employability is a vital resource that individuals aim to sustain or enhance (Acikgoz et al., 2016) and serves as a crucial factor in career development (Harari et al., 2023). Employability involves one’s perception of their ability to secure a new job, retain their current position, or obtain a better one (Hodzic et al., 2015). Understanding the mechanisms influencing employability has become a focal point in academia (Jabeen et al., 2022). Researching these mechanisms holds significant practical value for empowering individuals in their career choices and lifelong growth, enhancing organizational talent allocation and innovation, and promoting the release of social human-resource dividends and social fairness. Scholars have extensively researched the determinants of employability from various theoretical viewpoints. Individual factors such as proactive personality traits (Low et al., 2020), self-directed learning behaviors (Kim et al., 2015), career competencies (Blokker et al., 2019; Veld et al., 2015), career exploration (Praskova et al., 2015), and future time perspective (Froehlich et al., 2020) have been studied for their impact on employability. Additionally, environmental factors including career control (Veld et al., 2016), training and development activities (Veld et al., 2015), managerial support (Van Harten et al., 2016), coworker support (Karam et al., 2023), job demands (De Lange et al., 2020), job resources (Van Emmerik et al., 2012), organizational climate (Crans et al., 2021), and family support (Lo Presti et al., 2020) have been explored in relation to employability. Existing theories, such as JD-R and COR, primarily emphasize the balance between individual internal resources and needs or focus on social exchange relationships based on reciprocity. In contrast, signaling theory examines how information about future possibilities, conveyed by organizations to individuals, influences their attitudes and behaviors. This theory is particularly adept at explaining how individuals interpret signals, like organizational development measures, in employment settings characterized by information asymmetry and high uncertainty, subsequently adjusting their career psychology and behaviors.

While PSS predicts job seekers’ employability (Karimi et al., 2024; Xia et al., 2020), the underlying mechanisms warrant theoretical exploration. This study, rooted in Signaling Theory, introduces OS-E and PBE as mediating variables in the relationship between PSS and employability. Signaling theory suggests that when there is a lack of information symmetry, individuals communicate their worth to external entities (e.g., employers) through visible indicators (such as skills, assets, or characteristics), thus securing a competitive edge. Moreover, it can be applied to analyze the influence of PSS on one’s employability. PSS is defined as an individual’s subjective evaluation of external support (Zimet et al., 1988). In employment settings, social support signals influence self-perception, behavior, and resource acquisition. OS-E is one’s belief in completing work tasks (Schyns and Von Collani, 2002). This study acknowledges some overlap between the concepts of career self-efficacy and psychological capital. However, it emphasizes a focus on self-efficacy within specific career domains, which is more context-specific. Those with higher OS-E may proactively market themselves, transmitting quality signals to employers. Personal branding signals an individual’s quality, reducing information gaps with employers (Gorbatov et al., 2019), enhancing reputation, visibility, and recognition by employers (Gorbatov et al., 2024). In its classic formulation, signaling theory defines signals as market mechanisms whose credibility hinges on their cost, observability, resistance to imitation, and strategic intentionality (Spence, 1973). Signaling extends beyond brand attributes, and the existing framework captures only a portion of the signaling architecture outlined in the original theory. Through the lens of signaling theory, OS-E and PBE establish a signaling pathway linking psychological perception to market value, converting intangible social support into tangible employability. This mechanism elucidates the logical sequence from internal beliefs to outward image and, ultimately, to employment results, providing fresh perspectives on the development of employability.

## 2 Literature review and research hypothesis

### 2.1 Theoretical foundation

Signaling Theory, introduced by Nobel laureate Spence (Spence, 1973), elucidates that signal senders strategically convey credible behavioral cues to ensure accurate interpretation by signal receivers, thereby influencing their decision-making process. The essence of this theory lies in mitigating information gaps by unveiling concealed traits or information through signaling (Hopkins, 2012), encompassing both signaling and screening theories to offer a comprehensive framework for understanding information exchange and transactional behaviors in markets (Spence, 1973). Signaling Theory underscores that in recruitment scenarios where employers lack comprehensive candidate information, the party possessing superior information actively transmits signals to bridge the information gap (Connelly et al., 2011). Signaling theory suggests that individuals communicate discernible signals through particular behaviors or characteristics, which observers can interpret to impact assessments of their abilities. Within the labor market, job seekers’ PSS, OS-E, and PBE serve as crucial signaling sources. These signals play a pivotal role in shaping recruiters’ assessments and, consequently, influencing employability outcomes. In occupational and organizational psychology, social support is considered a vital personal and work resource, as outlined in the Job Demands-Resources (JD-R) theory. This theory, originally established by Demerouti et al., offers a meta-theoretical framework for examining how job characteristics affect employees’ health and motivation (Demerouti et al., 2001). Bakker and Demerouti expanded on this framework, categorizing social support as a work resource and detailing its dual roles: it fosters work engagement and growth by fulfilling psychological needs, and it supports mental health by reducing stress (Bakker and Demerouti, 2017). Research by Schaufeli and Bakker (2004) and others has further explored the “motivational process” mechanism, showing that resources can enhance long-term career development by boosting work engagement. Consequently, perceiving support as a resource can indirectly improve employability through motivational and health-protection processes, forming the theoretical basis of this study.

PSS denotes the tangible and emotional assistance individuals derive from their social circles, serving as a crucial social asset (Lakey and Orehek, 2011). Social support bolsters self-efficacy through vicarious experiences and verbal encouragement (Bandura, 1977). Individuals with heightened self-efficacy demonstrate proactive behavior in competitive job searches (Lent and Brown, 2006a) and exude confidence during interviews (Judge and Bono, 2001). The perception of external support translates into favorable psychological states, such as OS-E (behavioral signals) (Watanabe et al., 2025) and PBE (asset signals) (Gorbatov et al., 2019), thereby influencing employment-related behaviors and addressing employers’ concerns regarding “competence” and “trustworthiness.” Social support, while often beneficial, can sometimes act as a double-edged sword. Social support can generate information signals and potentially reinforce confirmation bias, self-enhancement bias, and the leniency effect. Consistent positive feedback from one’s social environment can bolster overly optimistic self-assessments and inflated perceptions of occupational self-efficacy. This feedback may also aid in building a personal brand that exceeds an individual’s actual productive capacity. Positive feedback from close relationships might include a tolerance filter that continually reinforces an individual’s overly optimistic self-perception, causing their professional self-confidence to deviate from reality. Simultaneously, it offers a low-cost means for image management. Individuals can leverage social networks to craft a professional image that emphasizes social skills over actual achievements. This relates to signaling theory, which posits that credible signals must incur costs. Although social support can enhance the quantity and strength of signals, it might also lower their cost, making them more prone to alteration or fabrication. Consequently, the distinction between the informational and persuasive nature of such signals becomes blurred, necessitating more rigorous scrutiny of their credibility by external observers.

**Figure 1:**
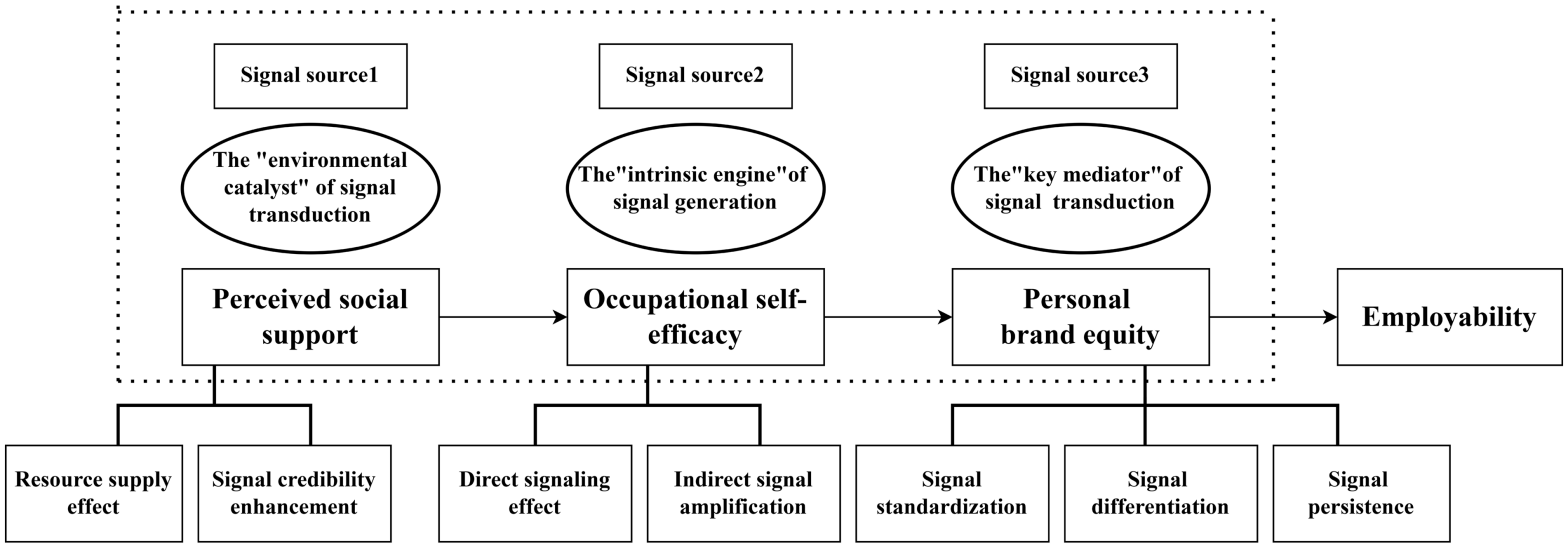
Research framework for this study.

While existing research on employability mechanisms predominantly delves into motivation and objectives, this study aims to develop an analytical framework grounded in Signaling Theory to delve deeper into the mechanisms and contextual limitations through which PSS impacts employability. PSS, depicted in Figure 1, functions as a environmental catalyst in the transmission of signals by providing individuals with subjective perceptions of supportive resources. Individuals with high PSS are more likely to access material and informational resources essential for career exploration, leading to increased firsthand success experiences and potentially heightened OS-E. Moreover, social support is associated with the credibility of PBE by acting as a third-party endorsement. Individuals with high core self-evaluations tend to interpret social support positively, resulting in heightened work engagement and organizational citizenship behaviors, reinforcing the positive signals of personal branding. OS-E, reflecting an individual’s confidence in their vocational abilities, plays a pivotal role as an intrinsic engine in signal generation. Individuals with high OS-E are inclined to undertake challenging tasks, inherently signaling competence. Furthermore, OS-E indirectly impacts employability by shaping PBE, which acts as a key mediator in signal transformation. PBE bridges OS-E and employability by translating abstract career competencies into tangible labels, thereby reducing information identification costs for recruiters. In a competitive job market, a distinctive personal brand aids job seekers in standing out. Additionally, PBE has a lasting cumulative effect, where stable signals contribute to sustainable enhancements in employability over time. In summary, the theoretical model of this study is illustrated in the figure. We hypothesize that perceived social support influences employability by sending two distinct signals. The first is a self-affirmation signal that enhances career self-efficacy, while the second is a value-highlighting signal to the market, which helps accumulate personal brand capital. These signals may function independently in parallel or have a chain-conduction relationship from the inside out. Together, they form a comprehensive signal-transmission mechanism through which social support impacts employability.

### 2.2 Research hypotheses

#### 2.2.1 The mediating role of occupational self-efficacy

Employability encompasses career identity, personal adaptability, social capital, and human capital (Fugate et al., 2004). Social support comprises objective and subjective components. PSS reflects an individual’s subjective perception of emotional care, instrumental assistance, or informational support from their social network (e.g., family, friends, colleagues), influencing psychological adaptation and behavior (Sarason et al., 1991). PSS, more than objective support, significantly impacts mental health (Uchino, 2009). Social Cognitive Career Theory (SCCT) views PSS as vital for navigating external environments (Zimet et al., 1988) and affecting work status (Lent et al., 1994). PSS not only offers employment resources but also bolsters employability through psychological and social pathways. Strengthening social support networks is positively associated with employability. Research shows PSS predicts job seekers’ resilience (Hou et al., 2024), work readiness (Tentama and Riskiyana, 2020), career decision self-efficacy (Cho et al., 2018; Guo, 2016; Restubog et al., 2010), and career maturity (Dietrich and Kracke, 2009; Turner et al., 2003), strengthening employability. Before examining the indirect path, this study will first investigate the direct relationship between the two variables. Thus, the following hypothesis is proposed:

*Hypothesis 1:* Perceived social support positively predicts employability.

Self-efficacy, as conceptualized by Bandura (1977), denotes an individual’s belief in their capacity to plan and execute actions required to attain goals in particular contexts. The notion of “OS-E” was initially introduced by Hackett and Betz in the vocational sphere, delineating it as an individual’s confidence in engaging in career-related behaviors, making educational and vocational choices, and persisting in these decisions (Betz and Hackett, 1997; Hackett and Betz, 1981). It reflects an individual’s belief in their ability to fulfill vocational responsibilities and meet professional expectations (Abele and Spurk, 2009). PSS serves to reinforce self-assessment by providing emotional and practical aid (Bandura, 1977), thereby augmenting self-efficacy. Individuals with elevated OS-E demonstrate proactive behavior in skill acquisition and professional networking, thereby enhancing their employability (Lent et al., 1994). Individuals with heightened PSS often exhibit increased resilience and confidence in addressing challenges across various domains. Social support offers emotional, informational, and practical resources, bolstering confidence in managing vocational obstacles (Hobfoll, 1989). Those with substantial PSS are more likely to perceive difficulties as surmountable hurdles, thus strengthening OS-E. In decision-making contexts, such individuals can effectively screen out adverse information that may impede problem-solving, leading to enhanced career decision-making performance. Positive interpersonal interactions, characterized by acceptance and respect, significantly enhance individuals’ optimism, thereby further fostering self-efficacy in career choices (Betz and Hackett, 2006). In light of the above, the study posits the following hypothesis:

*Hypothesis 2:* Perceived social support positively predicts occupational self-efficacy.

Bandura's (1977) self-efficacy theory posits that self-efficacy plays a crucial role in enhancing individual capabilities. OS-E, referring to an individual’s confidence in navigating job-seeking processes, is instrumental in bolstering employability, consequently leading to more successful behaviors. Employability is closely related to an individual’s self-efficacy (Mcdow and Zabrucky, 2015; Spurk et al., 2022; Ye et al., 2017). Strengthening self-efficacy not only facilitates securing better job opportunities but also fosters sustained career advancement. Individuals with heightened OS-E demonstrate increased confidence in their employability, resulting in improved job prospects, which, in turn, reinforces their beliefs in their OS-E (Berntson et al., 2008). OS-E emerges as a critical psychological catalyst for employability, directly influencing job-seeking behaviors and skill enhancement, while indirectly enhancing employment outcomes through improved adaptability and resource acquisition (Guan et al., 2015; Nilforooshan and Salimi, 2016). OS-E manifests in behaviors during interviews that employers perceive as indicators of job competence and growth potential. These behaviors include confidently showcasing past achievements, actively tackling complex tasks, proactively seeking feedback, and efficiently solving problems. Existing literature suggests that individuals with robust self-efficacy are adept at leveraging social resources (Seibert et al., 2001), setting ambitious career goals (Lent and Brown, 2006b), actively pursuing job opportunities (Kanfer et al., 2001), and demonstrating willingness to acquire new skills to meet labor market demands (Fugate and Kinicki, 2008). Moreover, high self-efficacy enables individuals to effectively cope with setbacks in job-seeking, reduce anxiety levels, and thereby sustain their employability (Schwarzer and Hallum, 2008). Building on this foundation, the present study formulates the following hypothesis:

*Hypothesis 3:* Occupational self-efficacy positively predicts employability.

Perceiving high levels of social support enhances individuals’ confidence in their abilities. Previous studies have shown that PSS positively influences job seekers’ psychological resilience and career adaptability (Hou et al., 2024). Individuals with higher resilience are less influenced by environmental stressors, interpreting stressful events as less threatening (Lent et al., 1994). They tend to approach challenges in the job search process rationally, thus boosting OS-E. Higher OS-E leads to active career exploration, proactive pursuit of job opportunities, and increased perseverance in the face of career-related obstacles (Judge and Bono, 2001). Individuals with strong OS-E demonstrate confidence in their employability, resulting in enhanced job prospects (Gong, 2014; Rothwell and Arnold, 2007). Social support furnishes resources that reinforce job seekers’ confidence, driving their job search behaviors and ultimately enhancing their employability (Bandura, 1977; Guan et al., 2013; Lent et al., 1994). By integrating prior research with Hypotheses 2 and 3, this study posits the following hypothesis:

*Hypothesis 4:* Occupational self-efficacy mediates the relationship between perceived social support and employability.

#### 2.2.2 The mediating role of personal brand equity

PBE not only reflects an individual’s internal psychological state but also serves as a crucial signal to the external job market, conveying one’s unique value and credibility. A personal brand encapsulates an individual’s unique traits, values, and beliefs, crafted into a distinct narrative and image with the goal of gaining a competitive edge among target audiences (Gorbatov et al., 2018). In today’s fiercely competitive job market, an increasing number of individuals actively curate their professional image and personal brand, drawing parallels to product branding strategies (Vallas and Cummins, 2015). PBE denotes the perceived value of a personal brand, hinging on its appeal, distinctiveness, and recognition within a specific professional sphere (Gorbatov et al., 2021; Gorbatov et al., 2024). Self-confidence and self-worth are pivotal factors in shaping brand image and projecting personal charisma within personal branding endeavors. A confident and esteemed personal brand is more likely to garner acknowledgment and trust, thus conferring a competitive advantage in career advancement and market competition. PSS fosters feelings of care, respect, and validation, significantly bolstering self-confidence and self-worth (Lent and Brown, 2013). Individuals perceiving strong social support tend to possess higher psychological capital, exhibit enhanced confidence, navigate decision-making with reduced social pressure, and demonstrate superior career judgment (Park et al., 2018). In professional settings, individuals with robust psychological capital often exhibit heightened self-efficacy and confidence, translating into improved work performance and outcomes that fortify their PBE. Building upon the aforementioned insights, this study posits the following hypothesis:

*Hypothesis 5:* Perceived social support positively predicts personal brand equity.

In the contemporary landscape of boundaryless careers, effective communication between employers and employees can be facilitated by actively transmitting signals to bridge information gaps, facilitating mutually beneficial outcomes for both parties (Ashuri and Bar-Ilan, 2017; Spence, 1973). Enhanced PBE in the job market leads to more comprehensive information availability, enabling firms to better assess an individual’s capabilities and attributes, thus conveying positive signals of competence (Gorbatov et al., 2024). Given firms’ inclination towards talent, their inherent information limitations, and the imperative to mitigate risks, individuals with higher PBE are more likely to secure employment, thereby bolstering their employability in the labor market (Dominique-Ferreira et al., 2022). Additionally, personal branding signals to employers that a job seeker is distinctive and capable of influencing their own and others’ perceptions through impression management strategies (Peng et al., 2023), subsequently impacting their popularity, perceived competence, compensation levels, and advancement pace within organizations (Fitriastuti and Vanderstraeten, 2022). Consequently, by cultivating an optimal professional self-image and nurturing PBE, individuals can garner superior performance evaluations and achieve favorable career outcomes, including enhanced job sustainability (Gorbatov et al., 2019). Thus, Hypothesis 6 is proposed:

*Hypothesis 6:* Personal brand equity positively predicts employability.

Research suggests that PSS furnishes individuals with emotional reinforcement, information conduits, and tangible assets, augmenting self-efficacy and career assurance (Hobfoll, 1989), while also aiding in establishing extensive social connections and fortifying social capital, thereby laying the groundwork for personal branding (Forret and Dougherty, 2004; Labrecque et al., 2011). PBE not only distinguishes individuals in job searches and career progression, attracting more professional opportunities, but also alleviates employers’ uncertainties regarding candidates’ competencies, thereby reducing career decision ambiguity and enhancing employability (Gorbatov et al., 2018, 2019; Minor-Cooley and Parks-Yancy, 2020). PSS offers individuals emotional reinforcement and practical assistance, reducing stress and enhancing self-assurance. This support serves as the foundation for developing PBE, which embodies refined social support resources in the form of professional competencies, communication skills, visibility, and reputation. PBE diminishes employers’ efforts in seeking information and conveys a proactive and ambitious image, ultimately improving employability and distinguishing individuals in the competitive job market. Consequently, the ensuing hypothesis is posited:

*Hypothesis 7:* Personal brand equity mediates the relationship between perceived social support and employability.

Through the lens of psychological empowerment, PSS boosts individuals’ self-assurance and effectiveness, thus nurturing the development of PBE (Demaray and Malecki, 2003; Zimet et al., 1988). This mechanism of psychological empowerment empowers individuals to demonstrate more initiative and creativity in their professional growth, thereby augmenting their employability. Enhanced subjective perceptions of external support correlate with increased self-efficacy in career decision-making (Ali et al., 2005). Individuals with heightened OS-E are more inclined to proactively overcome information barriers, effectively communicating clearer and more appealing messages to potential employers (Beck and Schmidt, 2012), shaping their personal brand, and cultivating a strong PBE, consequently enhancing their employability. Viewed from a signaling perspective, PBE functions as an indicator of individual competence, transmitting positive signals in the labor market and attracting a greater number of career prospects (Whitmer, 2019). This signaling mechanism amplifies the influence of social support on employability through PBE (Cable and Turban, 2003; Lair et al., 2005). From a resource transformation standpoint, PSS aids individuals in amassing PBE by providing resources, which is then transformed into career competitiveness, ultimately boosting employability (Cai et al., 2024; Khedher, 2019). Building upon the aforementioned points, this study posits the following hypothesis:

*Hypothesis 8:* Occupational self-efficacy and personal brand equity play a chain-mediating role in the influence of perceived social support on employability.

**Figure 2:**
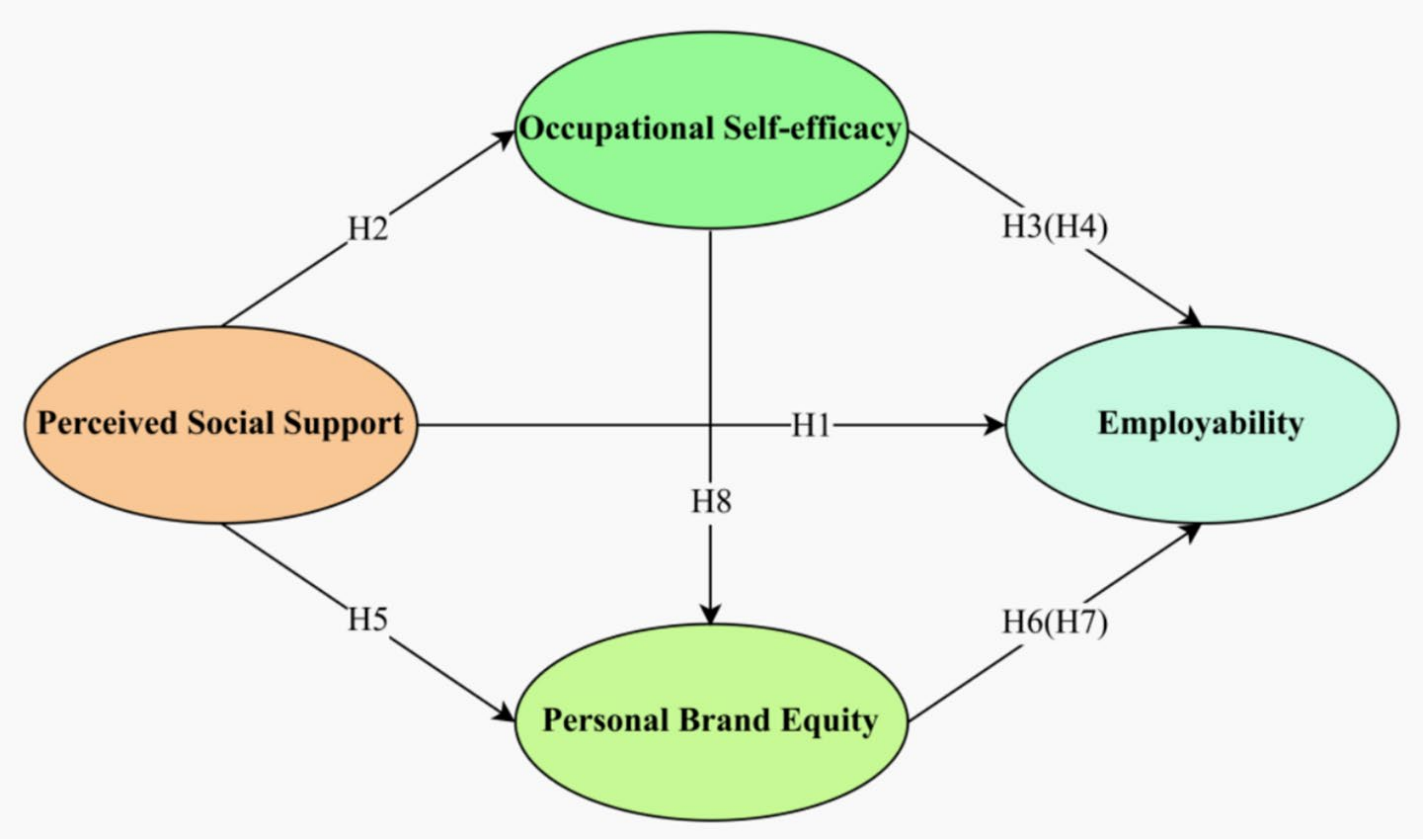
Hypothesis model.

This study introduces a novel chained mediation model that elucidates the relationship between PSS and employability through the lens of signaling theory. This model incorporates OS-E (an intrinsic confidence signal) and PBE (an extrinsic image signal). In labor markets characterized by information asymmetry, individuals must signal their unobservable abilities through observable cues. PSS, as an original resource, needs to be translated into two market-recognizable signals: self-efficacy (an intrinsic ability signal) and brand equity (an extrinsic value signal). Self-efficacy pertains to the perceived capability of “what I can do,” while brand equity relates to the perceived identity of “who I am.” The cultivation of personal brands is driven by self-efficacy, and feedback on the brand reinforces self-efficacy, collectively establishing a comprehensive personal signaling system that improves employers’ ability to decode information efficiently. This sequential logic chain delves into the mechanism through which PSS influences employability from a fresh perspective, as illustrated in the proposed research model below:

The hypothetical model is shown in Figure 2.

## 3 Materials and methods

### 3.1 Participants

In this study, a stratified cluster sampling method was utilized to conduct an online questionnaire survey of college students in China via the Wenjuanxing platform in March 2025. Stratification was primarily based on two dimensions: the region of the university (eastern, central, western) and the type of institution (“Double First-Class” universities, ordinary undergraduate universities, vocational colleges). This approach ensured the sample’s representativeness across regions and institutional levels. College classes served as cluster sampling units, with several classes randomly selected within each stratum. All students in these classes were invited to participate in the survey. During the survey’s execution, we collaborated with school staff to secure organizational support. The questionnaire instructions clearly communicated the research purpose, assured participants of complete anonymity, and stated that responses would be used solely for aggregate analysis without linking to personal identities. Participation was voluntary, based on informed consent. To prevent duplicate responses and maintain data quality, the following technical measures were implemented: (1) Participants provided their mobile phone numbers for unique identification; (2) A single-choice logical question at the questionnaire’s start screened out random responses; and (3) A response time threshold identified and excluded invalid responses. A total of 592 questionnaires were collected, with 58 deemed invalid and excluded, resulting in 534 valid questionnaires and an effective response rate of 90%. Regarding sample size adequacy, it is generally accepted that for structural equation model (SEM) analysis, the respondent-to-parameter ratio should be at least 10:1, with a recommended sample size over 200 (Tabri and Elliott, 2012). The final effective sample in this study was 534, with 47 parameters to be estimated, yielding a respondent-to-parameter ratio of approximately 11:1, thus meeting SEM analysis requirements.

### 3.2 Instruments

This study utilized established and validated scales to assess the variables. The three scales—the Perceived Social Support Scale, the Occupational Self-Efficacy Scale, and the Personal Brand Equity Scale—were originally in English. For this study, they were translated into Chinese using standard cross-cultural adaptation procedures. These Chinese versions have been thoroughly validated within the Chinese cultural context and exhibit strong psychometric properties. While a high Cronbach’s *α* coefficient (≥0.94) suggests strong item correlation, confirmatory factor analysis shows a clear factor structure for each scale. All items load on their respective factors at values above 0.6, with cross-loadings below 0.4. This demonstrates that the items, although reflecting the same construct, assess distinct aspects of it (e.g., family, friend, and others’ support in social support) rather than being redundant. The “translation-back translation” method (Brislin, 1970) was employed for English scales, with experts involved in the translation and back-translation process. Following a pilot survey, adjustments were made to scale items based on feedback from participants and expert evaluations. The reliability and validity of all scales were confirmed during the pilot survey. A 5-point Likert scale was used for all scales, with responses ranging from 1 for “strongly disagree” to 5 for “strongly agree.” We physically separated the scales for measuring core, mediating, and outcome variables in the questionnaire. Additionally, we partially balanced the presentation order of these scales to minimize the respondents’ mental set.

#### 3.2.1 Perceived social support

The research utilized the Multidimensional Scale of PSS (MSPSS), originally developed by Zimet et al. (1988) and subsequently revised by Chinese researcher Jiang (2001). This scale comprises 12 items assessing three dimensions: Family Support, Friend Support, and Significant Others Support, each consisting of 4 items. Higher scores indicate higher levels of PSS. The overall scale exhibited strong reliability, with a Cronbach’s alpha coefficient of 0.946. Additionally, the subscales demonstrated good internal consistency (α = 0.893 for Family Support, 0.871 for Friend Support, and 0.877 for Significant Others Support).

#### 3.2.2 Occupational self-efficacy

Rigotti et al. (2008) introduced a short version of the occupational self-efficacy scale to assess OS-E as a single-dimensional concept, comprising 6 items. Elevated scores reflect heightened OS-E in university students. The scale exhibited strong reliability in this investigation, with a Cronbach’s α coefficient of 0.943.

#### 3.2.3 Personal brand equity

Gorbatov et al. (2021) utilized the Personal Brand Equity Scale, which consists of 12 items categorized into Brand Appeal, Brand Differentiation, and Brand Recognition dimensions. The scale exhibited high reliability in this study, with a Cronbach’s α of 0.946. The internal consistency reliabilities for the subscales were 0.864, 0.934, and 0.960, respectively, indicating strong reliability.

#### 3.2.4 Employability

The research utilized Zhong’s College Students’ Employability Scale (Zhong, 2016), comprising 17 items categorized into five dimensions: critical thinking and forecasting, coordination and adaptability, teamwork and execution, communication skills, and professional competence. The overall Cronbach’s α coefficient for the scale was 0.962, indicating high internal consistency. Additionally, the subscales exhibited good reliability with internal consistency reliabilities of 0.956, 0.911, 0.920, 0.909, and 0.826 for the respective dimensions.

#### 3.2.5 Control variable

Previous studies have shown that demographic characteristics and learning experiences can impact individuals’ cognition and behavior in the context of OS-E and employability (Berntson et al., 2008). As such, this study included gender, age, household registration, only-child status, educational level, and discipline category as control variables.

### 3.3 Data analysis

This research utilized SPSS 26.0 and AMOS 29.0 for statistical analyses. Initially, the reliability and validity of the variables were assessed using both SPSS 26. and AMOS 29.0. Subsequently, descriptive statistics, correlation analysis, and hierarchical regression analysis were executed using SPSS 26.0. Lastly, the mediation effect was investigated through the PROCESS macro (model 6) in SPSS 26.0, employing the bias-corrected percentile Bootstrap method.

## 4 Results

### 4.1 Variable distinction validity tests

Before hypothesis testing, this study utilized AMOS 29.0 to conduct confirmatory factor analysis (CFA) on the model to assess the discriminant validity of the latent variables. Compared with other models, the four-factor model has a better goodness of fit. The results in Table 1 revealed that the four-factor model exhibited the most favorable fit statistics (χ^2^ /df = 3.273, CFI = 0.956, IFI = 0.956, TLI = 0.943, SRMR = 0.042, RMSEA = 0.069) among the various models tested, signifying strong discriminant validity of the measurement model. In SEM, a variety of goodness-of-fit indices are used to thoroughly assess a model’s fit. For χ^2^/df, a value under 3 signifies a good fit, while a value under 5 is deemed acceptable; smaller values indicate better fit. Both CFI and TLI require values above 0.95 for a good fit and above 0.90 for an acceptable fit, with values closer to 1 indicating better model fit. An SRMR value below 0.08 is necessary for a good fit, with lower values being preferable. For RMSEA, a value under 0.06 is considered good, and under 0.08 is acceptable; smaller values suggest less approximation error between the model and the data. Together, these indices form the fundamental criteria for evaluating the goodness of fit in structural equation models.

**Table 1:** Confirmatory factor analysis results.

Models	χ^2^/df	CFI	TLI	SRMR	RMSEA
Four-factor model + CMV	2.231	0.980	0.969	0.039	0.051
Four-factor model: PSS, OS-E, PBE, E	3.273	0.956	0.943	0.042	0.069
Three-factor model: PSS, OS-E + PBE, E	8.122	0.847	0.820	0.074	0.122
Two-factor model: PSS + OS-E + PBE, E	10.868	0.784	0.751	0.099	0.144
One-factor model: PSS + OS-E + PBE + E	15.107	0.689	0.644	0.104	0.172

CMV denotes common method variance, PSS represents perceived social support, OS-E stands for occupational self-efficacy, PBE indicates personal brand equity, and E refers to employability.

### 4.2 Common method bias test

This study utilized Harman’s single-factor test to assess common method bias. The unrotated exploratory factor analysis indicated that the initial factor explained 34.9% of the variance, falling below the critical threshold of 40% (Zhou and Long, 2004). Additionally, a common method variance (CMV) factor was integrated into a five-factor model. If the original confirmatory factor analysis (CFA) model with correlated factors demonstrates notable improvements in fit indices following the addition of a method factor (e.g., CFI and TLI increases exceeding 0.1, RMSEA and SRMR decreases surpassing 0.05), it suggests the presence of substantial common method bias. Results presented in Table 1 demonstrated that the inclusion of the CMV factor did not contribute to a significant enhancement in model fit compared to the four-factor model (Δχ^2^/df = 1.042, ΔCFI = 0.024, ΔTLI = 0.026, ΔSRMR = 0.003, ΔRMSEA = 0.018), with all metrics remaining within acceptable ranges (Wen et al., 2018). Consequently, no notable common method bias was identified in this study, enabling further statistical analyses.

### 4.3 Descriptive statistics and correlation analysis

Table 2 displays the means, standard deviations, and correlation coefficients among the variables. The correlation analysis reveals significant positive correlations between PSS, OS-E, and PBE with employability (*r* = 0.636, *p* < 0.01; *r* = 0.727, *p* < 0.01; *r* = 0.726, *p* < 0.01). Moreover, significant associations are evident between PSS and both OS-E and PBE (*r* = 0.661, *p* < 0.01; *r* = 0.519, *p* < 0.01; *r* = 0.696, *p* < 0.01). This suggests that psychological and social resources are interconnected and collectively play a crucial role in influencing employability. The correlations among the variables align with the hypotheses, offering preliminary support for them. While some variables exhibit relatively high correlation coefficients (> 0.70), potentially raising concerns about multicollinearity, the variance inflation factor (VIF) test in the subsequent regression analysis reveals that all variables have VIF values below the critical threshold of 5. This indicates that multicollinearity does not significantly affect the model estimation.

**Table 2:** Descriptive statistics and correlations of all variables (*n* = 534).

Items	1	2	3	4	5	6	7	8	9	10	11	12	13	14	15
1. Perceived social support	1														
2. Friend support	0.923**	1													
3. Family support	0.927**	0.790**	1												
4. Significant others support	0.921**	0.785**	0.766**	1											
5. Occupational self-efficacy	0.661**	0.637**	0.599**	0.598**	1										
6. Personal brand equity	0.519**	0.477**	0.476**	0.486**	0.696**	1									
7. Brand appeal	0.626**	0.586**	0.567**	0.583**	0.779**	0.811**	1								
8. Brand differentiation	0.487**	0.449**	0.445**	0.457**	0.653**	0.945**	0.745**	1							
9. Brand recognition	0.304**	0.270**	0.285**	0.285**	0.452**	0.872**	0.474**	0.749**	1						
10. Employability	0.636**	0.631**	0.543**	0.596**	0.727**	0.726**	0.786**	0.675**	0.497**	1					
11. Professional competence	0.502**	0.488**	0.452**	0.454**	0.654**	0.754**	0.695**	0.696**	0.612**	0.839**	1				
12. Communicative skills	0.611**	0.602**	0.531**	0.567**	0.643**	0.601**	0.709**	0.558**	0.370**	0.905**	0.701**	1			
13. Teamwork and execution	0.590**	0.599**	0.487**	0.558**	0.618**	0.538**	0.709**	0.488**	0.285**	0.903**	0.647**	0.852**	1		
14. Coordination and adaptability	0.589**	0.572**	0.488**	0.577**	0.656**	0.626**	0.689**	0.589**	0.415**	0.911**	0.672**	0.781**	0.816**	1	
15. Critical thinking and forecasting	0.512**	0.523**	0.433**	0.471**	0.631**	0.675**	0.663**	0.640**	0.502**	0.849**	0.670**	0.657**	0.672**	0.744**	1
Mean	3.658	3.712	3.655	3.607	3.576	3.241	3.592	3.216	2.914	3.539	3.375	3.630	3.716	3.601	3.371
SD	0.670	0.694	0.804	0.764	0.674	0.658	0.638	0.747	0.862	0.590	0.682	0.672	0.650	0.677	0.664
Skewness	0.140	0.082	−0.098	0.063	0.358	0.680	0.282	0.455	0.174	0.447	0.449	0.116	0.094	0.253	0.576
kurtosis	−0.084	0.106	−0.156	−0.053	0.420	1.515	0.816	1.077	0.829	0.764	1.033	0.215	0.012	0.270	0.579

*n* = 534; ** *p* < 0.01.

### 4.4 Hypothesis testing

This study utilized hierarchical regression analysis and the Bootstrap mediation test to evaluate the research hypotheses comprehensively. As detailed in Table 3, after accounting for demographic variables like gender and age, perceived social support significantly predicted career self-efficacy (Model 1: *β* = 0.627, *p* < 0.001, *R*^2^ = 0.448, *F* = 53.212, *p* < 0.001), personal brand equity (Model 2: *β* = 0.485, *p* < 0.001, *R*^2^ = 0.290, *F* = 26.758, *p* < 0.001), and employability (Model 4: *β* = 0.536, *p* < 0.001, *R*^2^ = 0.414, *F* = 46.273, *p* < 0.001), thus supporting Hypotheses H1, H2, and H5. Further analysis showed that career self-efficacy (Model 6: *β* = 0.634, *p* < 0.001, *R*^2^ = 0.533, *F* = 74.903, *p* < 0.001) and personal brand equity (Model 7: *β* = 0.647, *p* < 0.001, *R*^2^ = 0.533, *F* = 74.685, *p* < 0.001) independently predicted employability, affirming Hypotheses H3 and H6. In the comprehensive model (Model 8: *R*^2^ = 0.656, *F* = 99.878, *p* < 0.001), which included perceived social support, career self-efficacy, and personal brand equity as predictors of employability, all three variables retained significant positive predictive effects (perceived social support: *β* = 0.200, *p* < 0.001; career self-efficacy: *β* = 0.259, *p* < 0.001; personal brand equity: *β* = 0.358, *p* < 0.001). Additionally, the direct effect of perceived social support on employability was notably weaker than in Model 4, indicating that career self-efficacy and personal brand equity mediated this relationship (Wen et al., 2004). This finding was corroborated by the Bootstrap test using the Process program (with 5,000 resamples), confirming significant mediation effects and validating Hypotheses H4, H7, and H8. In summary, hierarchical regression analysis and the mediation effect test demonstrated that perceived social support not only directly predicts employability but also indirectly influences it through the mediating roles of career self-efficacy and personal brand equity. The final model accounted for 65.6% of the variance in employability, indicating strong predictive power.

**Table 3:** Multilayer linear regression analysis results (*n* = 534).

Variables	Occupational self-efficacy	Personal brand equity		Employability			
	Model 1	Model 2	Model 3	Model 4	Model 5	Model 6	Model 7
Genders	−0.095*	−0.049	0.008	−0.022	0.023	−0.015	0.02
Age	0.024	0.055**	0.038*	0.013	−0.004	−0.026	−0.013
School level	0.134	0.181	0.128	0.111	0.109	0.127	0.011
Grade	−0.034	−0.093**	−0.069*	−0.041	−0.015	0.026	0.002
Census register	−0.015	−0.063	−0.042	−0.082	−0.05	−0.006	−0.056
Only child	−0.051	−0.047	−0.022	−0.054	−0.041	−0.055	−0.024
Degree type	0.004	0.002	0.024	−0.001	−0.002	−0.001	−0.003
Perceived social support	0.627***	0.485***		0.536***			0.2***
Occupational self-efficacy			0.673***		0.634***		0.259***
Personal brand equity						0.647***	0.358***
R2	0.448	0.290	0.492	0.414	0.532	0.533	0.656
△R2	0.399	0.250	0.453	0.382	0.501	0.502	0.080
*F*	53.212***	26.758***	63.590***	46.273***	74.685***	74.903***	99.878***

* *p* < 0.05, ** *p* < 0.01, *** *p* < 0.001.

The statistical analysis utilized the SPSS macro program Process, developed by Hayes. The Bootstrap method was applied for resampling the sample 5,000 times, facilitating the analysis of the mediation effect and conducting a significance test. The study revealed a significant total effect of PSS on employability (Effect = 0.538, SE = 0.028, 95% CI [0.483, 0.594]), as well as a significant direct effect (Effect = 0.199, SE = 0.029, 95% CI [0.142, 0.256]), supporting Hypothesis H1. Consistent with previous research (Mackinnon et al., 2004), the confirmation of the indirect effect was based on the non-inclusion of zero within the confidence interval. Path 1 exhibited an effect size of 0.164 (95% CI [0.109, 0.223]), explaining 30.5% of the total effect and indicating a substantial mediating effect of OS-E in the relationship between PSS and employability, thus confirming Hypothesis H4. Path 2 showed an effect size of 0.036 (95% CI [0.008, 0.067]), accounting for 6.7% of the total effect, supporting a significant mediating effect of PBE on the relationship between PSS and employability, validating Hypothesis H7. The combined effect of OS-E and PBE in the chain mediation analysis was 0.140 (95% CI [0.101, 0.182]), explaining 26.0% of the total effect and affirming the crucial mediating roles of these factors between PSS and employability, thereby confirming Hypothesis H8. The statistical analysis outcomes generated by the PROCESS macro (model 6) can be found in Table 4.

**Table 4:** Bootstrap analysis of mediating effect significance test.

Name	Effect value	Boot SE	95% confidence interval		*p*	Effect ratio (%)
			Boot LLCI Boot ULCI			
Total effect	0.538	0.028	0.483	0.594	0.000	/
Direct effect	0.198	0.029	0.142	0.256	0.000	36.8
Total indirect effect	0.340	0.032	0.278	0.403	0.000	63.2
Path 1	0.164	0.029	0.109	0.223	0.000	30.5
Path 2	0.036	0.015	0.008	0.067	0.000	6.7
Path 3	0.140	0.021	0.101	0.182	0.000	26.0

Path 1: Perceived Social Support – Occupational Self-efficacy – Employability; Path 2: Perceived Social Support – Personal Brand Equity – Employability; Path 3: Perceived Social Support – Occupational Self-efficacy – Personal Brand Equity – Employability.

**Figure 3:**
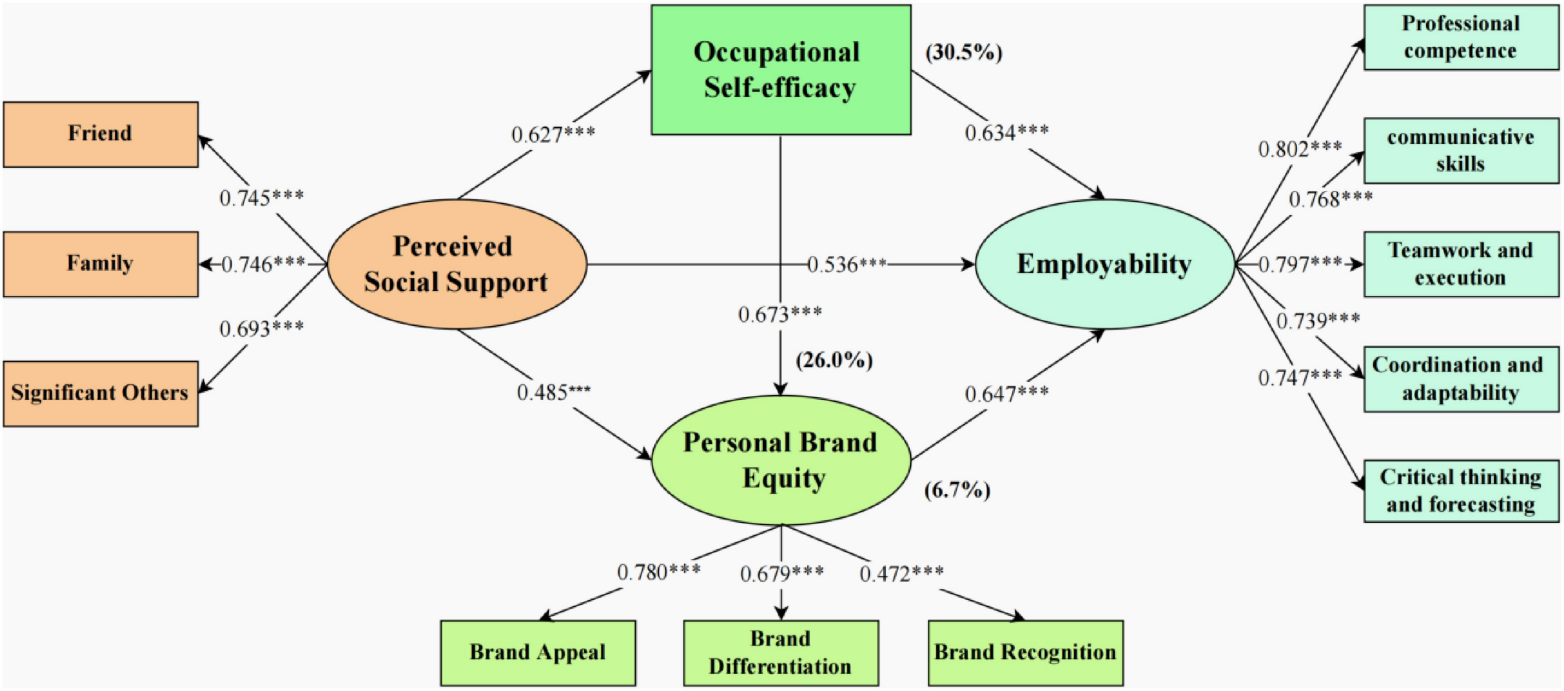
The theoretical model results of this study. *** *p* < 0.001. The proportion of indirect effect is already in the parentheses.

The theoretical model outcomes are depicted in Figure 3, displaying the path coefficients among variables as detailed below.

## 5 Discussion

Drawing on signaling theory, this research delves into the impact of PSS on individual employability by examining two crucial mediating factors: OS-E and PBE. The empirical findings robustly corroborate the research hypothesis, elucidating the inherent mechanism through which individuals’ PSS influences their employability. OS-E, centered on internal drive and actions, forms the bedrock for proactive behavior. Conversely, PBE underscores external portrayal and market acknowledgment, playing a pivotal role in realizing value. A potential symbiosis between the two is conceivable: heightened self-efficacy fosters more effective brand establishment endeavors, while favorable brand reception further bolsters self-efficacy. This synergistic interplay collectively delineates the comprehensive pathway through which social support amplifies employability. This mechanism underscores that social support not only furnishes resources but also facilitates the dissemination of positive signals in the job market through psychological impetus and behavioral direction.

### 5.1 Theoretical enlightenment

This study leverages signaling theory to reveal how perceived social support influences job seekers’ employability through the lens of information asymmetry. It also broadens the application of signaling theory within employment research (Spence, 1973). While existing literature primarily examines employability antecedents through frameworks such as social exchange, conservation of resources, and job demands-resources (JD-R) (Clements and Kamau, 2018; Jia et al., 2024; Solberg and Dysvik, 2016), these approaches primarily focus on social support as a direct resource or buffer. They often overlook how individuals convert received support into competitive advantages recognized in the labor market, especially under conditions of information asymmetry. This study addresses this gap by applying signaling theory. OS-E serves as an internal belief signal regarding one’s capabilities developed through processing social support signals (Betz and Hackett, 1981), while PBE represents the reputation signal crafted in the external labor market through the behavioral adaptation of social support signals (Baker et al., 1994). The research indicates that high OS-E drives individuals to engage actively in brand-building activities, enhances resilience against negative feedback, and facilitates resource accumulation. Job seekers with high self-efficacy attract more career opportunities and social capital through confident behaviors, enriching their personal brand. This “psychological signal – behavioral signal – market outcome” chain underscores the proactive signal-management role of job seekers in career management, a dimension that theories like JD-R or COR, which emphasize environmental and resource interactions, often overlook.

This study introduces OS-E and PBE as mediating variables to clarify how PSS influences employability, integrating psychological and social perspectives in employability research. This study elucidates the roles of OS-E and PBE as crucial psychological and social mechanisms in translating PSS into employability. By advancing beyond prior research that predominantly examined direct effects of social support or single mediators, this study introduces a comprehensive and nuanced chain mediation model, enhancing the theoretical understanding of factors influencing employability. PSS boosts OS-E, encouraging individuals to actively develop their personal brands, thus enhancing employability. This mediation chain illustrates the pathway of social support on employability through psychological, behavioral, and outcome stages (Bandura, 1977; Ehrhart and Ziegert, 2005; Labrecque et al., 2011). The results indicate that improving employability necessitates robust internal resilience (self-efficacy) and effective external presentation and networking (PBE), with social support fostering both aspects. Moreover, by emphasizing PSS, this study reaffirms the critical role of individual subjective perception in signal reception and transformation processes. Identical supportive behaviors may be interpreted and utilized with varying efficiency among individuals, elucidating reasons for differences in employability performance within similar social networks.

While this study primarily confirms that perceived social support shows a positive relationship with employability by boosting self-efficacy and personal brand value, as suggested by signaling theory, it’s crucial to adopt a more balanced view. Although signals help reduce information asymmetry, they can also lead to performativity risks and internal alienation. This may transform personal brand building into a strategic response to external expectations, causing anxiety, self-objectification, and harm to long-term career identity. This study primarily focuses on job seekers and employees within specific cultural contexts. Cultural values can shape how individuals perceive social support, express self-efficacy, and approach personal brand building. Additionally, when applying these research findings to groups at various developmental stages, in different industries, or with diverse socioeconomic backgrounds, their generalizability requires further validation. Future research should encompass a wider array of cultures and sub-groups.

### 5.2 Implications in practice

Prioritizing the establishment of social support networks and enhancing job seekers’ proficiency in “signal sending” are crucial initial steps. PSS, functioning as an implicit signal, significantly impacts individuals’ employability (Spence, 1973), mitigating negative emotions and boosting confidence in navigating job search challenges (Wanberg et al., 2000). Job seekers should not only emphasize hard skills but also acknowledge the importance of “PSS,” “OS-E,” and “PBE “as vital “soft skills” that communicate positive signals to potential employers (Gorbatov et al., 2024; Khedher, 2019; Zhang et al., 2023). Encouraging job seekers to actively seek and leverage support from family, friends, mentors, alumni, and professionals is essential (Adler and Kwon, 2002). Families should provide emotional support, while schools must offer internships and career guidance. Additionally, society should develop training and employment support systems. A collaborative network involving the government, universities, enterprises, and families should be established to collectively improve job seekers’ competitiveness and career clarity.

Prioritizing the enhancement of OS-E is crucial for systematically developing PBE. OS-E acts as a vital psychological mechanism that links PSS to employability outcomes (Kanfer et al., 2001). Universities and enterprises should assist job seekers in gaining successful experiences and establishing role models through training, coaching, and other methods to boost their OS-E. Strong OS-E forms the basis for job seekers to communicate a confident “I can do it” message to potential employers. PBE, serving as an “observable signal” in the job market, directly impacts employability (Anderson and Tomlinson, 2021; Spence, 2002). A robust PBE helps job seekers stand out in the job market, increasing their chances of gaining employer recognition (Gorbatov et al., 2024). Job seekers should enhance their proactive job-search behaviors by adopting a correct and positive attitude towards challenges. They should actively seek support, identify and utilize resources, and turn these into motivational factors to deepen their career exploration and clarify their future professional identity (Taber and Blankemeyer, 2015). Additionally, job seekers need to cultivate a “sense of urgency,” define clear self-positioning and career planning, prioritize career development (Zheng and Chang, 2023), and consciously enhance their career efficacy to improve their professional self-image and develop stronger PBE.

Addressing information asymmetry between employers and job seekers is crucial through the establishment of efficient signaling and verification mechanisms (Bergh et al., 2019). Job seekers can enhance their personal brand signals by translating social support into tangible professional achievements, thereby mitigating information barriers in resume screening and reducing employers’ decision-making risks at a low cost (Gorbatov et al., 2018). Additionally, cultivating grit, characterized by perseverance and passion for long-term goals, is vital for demonstrating sustained endurance and achieving success in a specific task or career (Duckworth et al., 2007). Developing grit enables job seekers to navigate challenges, enhance skill development, and gain a competitive edge in their field, thereby amplifying their signal value and intensity (Gonlepa et al., 2023). Employers, on the other hand, should actively support job seekers in building their PBE (Backhaus and Tikoo, 2004). This involves fostering communication, promoting OS-E, providing training in psychological resilience and job-specific skills, and offering increased support to employees with high OS-E. Concurrently, job seekers should proactively showcase their talents, capabilities, and innovative ideas to bridge the information gap between themselves and potential employers.

### 5.3 Limitations and future directions

This study is subject to several limitations. Primarily, the research sample is constrained in its scope. The investigation delves into the employment hurdles encountered by college graduates, exploring the interplay among PSS, OS-E, PBE, and employability. Employability, a fluid concept, undergoes continual development, refinement, and enhancement. Individuals in diverse life stages, environments of growth, and occupational realms may manifest differing levels of PSS, OS-E, and PBE (Duggan et al., 2022). Discrepancies in PSS, OS-E accumulation, and PBE are evident among college students across various academic years. The impact of PSS on employability necessitates further substantiation. Subsequent research should broaden the participant pool to contrast employability variances among cohorts at distinct developmental phases and professional categories, thereby bolstering the research findings’ credibility.

Further refinement of the research methodology is warranted. This study utilized cross-sectional data to examine the interplay among variables. While the collected data successfully passed the common method bias test, the reliance on self-reported measures for all variables introduced inherent subjectivity. To enhance the study’s reliability and validity, future research could consider employing the triangulation method. This approach involves gathering data from diverse perspectives or viewpoints at various time points to offer a more comprehensive and objective assessment of variables (Jick, 1979). To elucidate the causal link between PSS and employability, future studies could enhance research design. Future research could be enhanced by employing multi-source and multi-method measurement approaches to improve the external and ecological validity of the findings. Firstly, we will employ experimental methods to gather longitudinal data at various time points for follow-up research. This approach will allow us to rigorously verify the mediating mechanism we initially identified. Secondly, leveraging qualitative research techniques such as interviews could provide deeper insights into the mechanisms of influence, thereby bolstering the research findings’ credibility.

Further investigation is needed to explore the connections between variables. Drawing on signaling theory, this research delves into two mediating factors: OS-E and PBE. Findings suggest that OS-E and PBE do not completely mediate the impact of PSS on employability. Subsequent studies could take a multi-theoretical approach by integrating essential factors like proactive personality, organizational management strategies, and future time perspective (Jiang et al., 2023) to enhance understanding of how university students’ PSS affects their employability through various pathways and under different circumstances. The conceptual model of this study primarily elucidates the role of social support as a resource for signal generation. While we theoretically acknowledged its potential bias effects, we did not explicitly model the boundary conditions and outcome variables associated with these distortions. For example, under what network structures or cultural contexts is social support more apt to produce informative rather than distortive signals? What are the long-term career consequences of distorted signals? These questions represent crucial avenues for future research.

## Data Availability

The original contributions presented in the study are included in the article/supplementary material, further inquiries can be directed to the corresponding author.
